# Treating metastatic extraocular retinoblastoma complicated with Langerhans cell histiocytosis

**DOI:** 10.3205/oc000258

**Published:** 2025-11-04

**Authors:** Marya Hameed, Fatima Siddiqui, Muhammad Khuzzaim Khan, Sindhura Tadisetty, Prasanna Kumar Gangishetti

**Affiliations:** 1 National Institute of Child Health, Karachi, Pakistan; 2 Dow University of Health Sciences, Karachi, Pakistan; 3 University of Kentucky, Lexington KY, USA

**Keywords:** low vision, chemotherapy, eye neoplasms, Langerhans cell histiocytosis, retinoblastoma

## Abstract

**Objective:**

Retinoblastoma (Rb) and Langerhans cell histiocytosis (LCH) are rare and distinct diseases that can coexist in a patient. We present a case report of a 5-year-old male who was diagnosed with bilateral retinoblastoma and LCH involving the skull and spine.

**Methods:**

The patient underwent a detailed clinical evaluation, including a complete ophthalmic examination, neuroimaging studies, and bone marrow biopsy. A genetic test confirmed the presence of the BRAF V600E mutation in the LCH lesion. Treatment with BRAF inhibitors was initiated for LCH, followed by chemotherapy and left eye enucleation for retinoblastoma. The patient was monitored closely during treatment and at follow-up visits.

**Results:**

The patient responded well to therapy, with no evidence of disease recurrence at 12-month follow-up and the enucleated eye was replaced with a prosthesis. The BRAF inhibitor was found to be an effective therapeutic option for the patient with BRAF-positive LCH.

**Conclusion:**

Our case highlights the importance of early diagnosis and prompt treatment in managing complex cases with coexisting retinoblastoma and LCH. Treatment with BRAF inhibitors could be a promising therapeutic option for patients with BRAF-positive LCH. Further studies are needed to evaluate the efficacy and safety of BRAF inhibitors in the treatment of LCH. The long-term outcome and potential late effects of combined therapy for coexisting retinoblastoma and LCH should also be monitored closely.

## Introduction

Retinoblastoma is a rare but significant malignancy in children, originating from primitive retinal cells [1], [2]. It is caused by mutations in the Rb gene, which is a tumor suppressor gene located on chromosome 13q14, or MYCN amplification [3]. The worldwide reported incidence of Rb is 1 in 16,000 to 18,000 live births [4], [5]. The tumor has historical significance for inspiring the “two-hit hypothesis” for oncogenesis [5] and being the first cancer to have its hereditary component added to its AJCC TNM classification [6], [7]. Although Rb has a good prognosis with a five-year survival rate of around 90%, it is an aggressive tumor that can spread extra-ocularly, making early detection and treatment essential.

Langerhans cell histiocytosis (LCH), previously known as histiocytosis X, is a relatively uncommon condition characterized by the proliferation of myeloid dendritic cells expressing CD1a, the same antigen expressed in Langerhans cells in the skin [8], [9]. LCH is observed more commonly in infants and children.

In this report, we present a case initially diagnosed as LCH due to multiple skull-based lesions and positive bone biopsy. However, subsequent imaging revealed metastatic intracranial retinoblastoma with extensive leptomeningeal seeding and skull-based lesions. This case highlights the importance of considering metastatic retinoblastoma as a differential diagnosis in cases of LCH with bone lesions and the significance of early detection and treatment of Rb. Our report can guide clinicians and radiologists in recognizing this rare and complex presentation of Rb with LCH.

## Case description

A 5-year-old boy was brought to the pediatric radiology department due to complaints of progressively enlarging swellings on his head over the past 3 years. He also reported experiencing headaches, vomiting, and associated exophthalmos and prominent proptosis of the left eye, with compromised vision in that eye, as reported by his mother. There was no history of fever, respiratory infection, or trauma.

**Figure 1:**
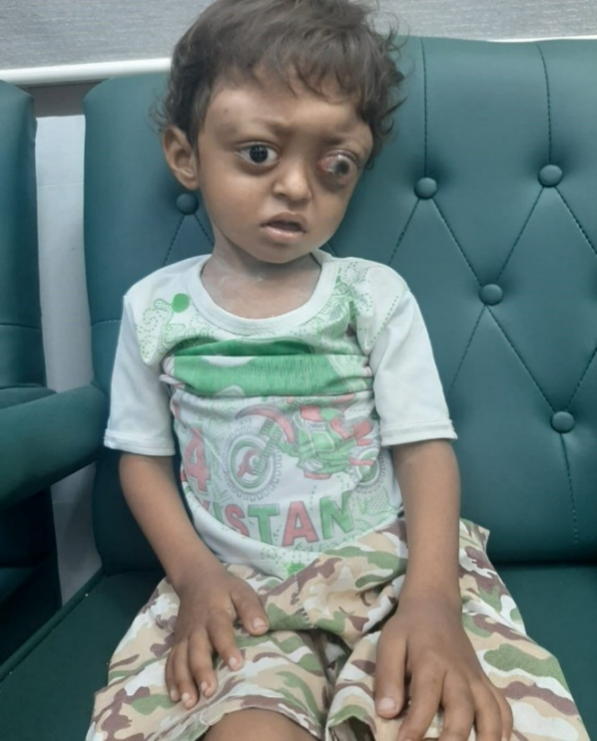
Image of the patient with left-sided retinoblastoma and multifocal LCH skull lesions

Systemic examination revealed multifocal skull deformities and gait difficulty. Ophthalmic examination showed left-sided leukocoria, decreased visual acuity without light perception, and +1 anterior chamber flare (Figure 1 (Fig. 1)). Blood workup was performed which revealed normal hemoglobin levels of 12 mg/dl, a white blood cell count within the normal range of 8,000/mm^3^ and platelet count of 300/000x10^9^/L. Liver function tests demonstrated mildly elevated levels of aspartate aminotransferase (AST) a 45 U/L and alanine aminotransferase (ALT) at 32 U/L, while total bilirubin and alkaline phosphatase levels were within normal limits. Renal function tests, including blood urea nitrogen (BUN) and creatinine, showed no abnormalities. Inflammatory markers such as C-reactive protein (CRP) and erythrocyte sedimentation rate (ESR) were elevated at 50 mg/L and 29 mm/h, respectively, indicating an ongoing inflammatory process. Calcium and phosphorus levels were within normal ranges, and tumor markers, including alpha-fetoprotein (AFP) and beta-human chorionic gonadotropin (β-hCG), were also normal. 

**Figure 2:**
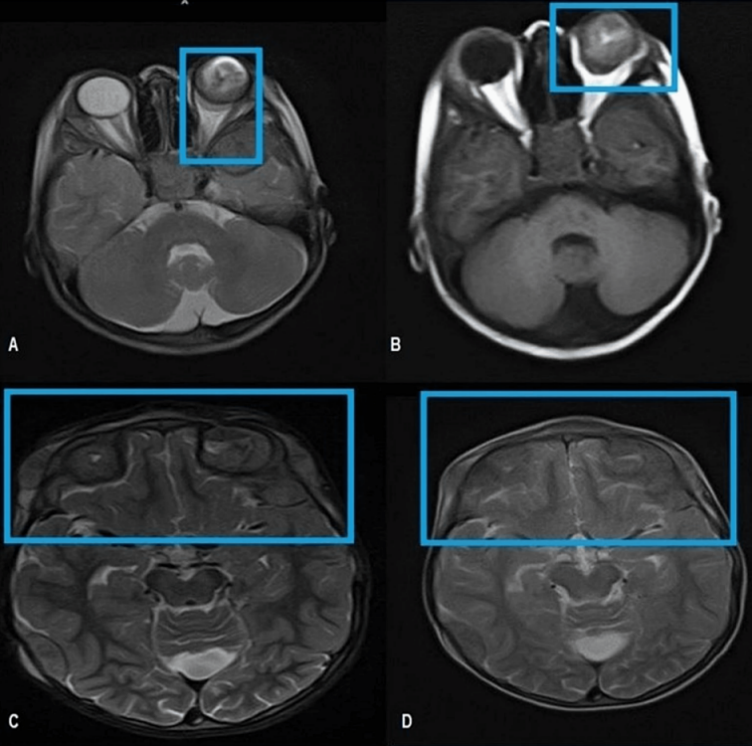
Enlarged left orbit showing intraocular retro globular mass with retinal detachment and T1W bright signal suggesting internal hemorrhage. A: T2W Axial MRI brain reveals an intraocular mass in the posterior aspect of the left globe abutting the retina returning heterogeneous hypointense signals. B: T1W axial MRI brain shows an intraocular lesion with a V-shaped T1W hyperintense signal in the posterior left globe reflecting hemorrhagic retinal detachment. Note the extraocular extension of the tumor through the sclera resulting in the deformed globe. C and D: Axial MRI T2W images cranial section reveals extra-axial low signal intensity masses in the bi-frontal region and left scalp.

**Figure 3:**
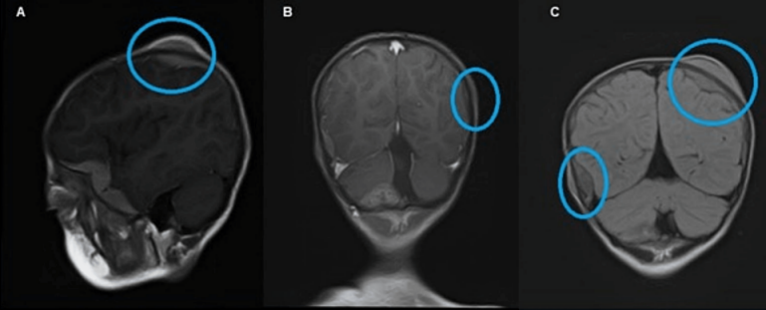
MRI showing multifocal scalp-based lesions with enhancing extradural soft tissue component seen along the left greater wing of sphenoid, bifrontal, and left parietal region producing bulge and mild leptomeningeal enhancement. Sagittal MRI (A), post-gadolinium coronal MRI (B), and FLAIR (C) show multifocal scalp-based lesions with enhancing extradural soft tissue components seen along the left greater wing of sphenoid, left high parietal, and left occipital region.

Magnetic resonance imaging (MRI) of the brain and orbit showed an intraocular retro-globular mass with retinal detachment and orbital hemorrhage. There were multifocal scalp-based lesions with an enhancing extradural soft tissue component along the left greater wing of the sphenoid, bi-frontal, and left parietal regions, causing bulges and mild leptomeningeal enhancement (Figure 2 (Fig. 2) and Figure 3 (Fig. 3)).

A lumbar puncture was performed, which showed clumps of malignant cells, confirming the imaging suspicion of diffuse infiltrating stage IV-B retinoblastoma with cerebrospinal fluid (CSF) metastasis. Biopsies of the thickened areas in the skull and local skin were taken, and a diagnosis of LCH was made based on the presence of proliferative Langerhans cells and eosinophils with positive staining for S-100 and CD207. BRAF V600E genotyping and MAP2K1 sequencing were performed, and an activating somatic mutation was found in the BRAF V600E gene. The child was referred to the pediatric oncology department, where treatment was planned. He was started on carboplatin/vinblastine/etoposide and the BRAF inhibitor Vemurafenib on alternative cycles for 1 year for retinoblastoma and histiocytosis, respectively, along with radiation therapy of CSF. The retinoblastoma therapy was administered through intra-arterial and intra-thecal routes. Follow-up testing performed one and three months post-treatment showed that his CSF was cancer-free, and enucleation of the left eye was performed. He was then started on three months of vinblastine/prednisone/mercaptopurine. The skull swellings significantly decreased, and the treatment was discontinued. The patient was put on remission follow-ups for 5 years. Until one year after treatment, the patient has not shown any signs of recurrence.

Written and signed consent was obtained from the parents for the production and publication of this case report. The National Institute of Child Health’s radiology head of the department gave ethical approval for the publication of figures and the case.

## Discussion

Retinoblastoma is a rare and aggressive tumor that affects the eye and can metastasize to other parts of the body, including the lymph nodes, CNS, and abdomen. In this case, the patient had Stage IV-B retinoblastoma with CSF metastasis, which is a rare and advanced form of the disease. The incidence of metastases in retinoblastoma is relatively low, with a reported incidence of 4.8% in the USA [10]. However, when metastases do occur, they most commonly involve the CNS, accounting for 50% of cases [11]. The risk of CNS metastases is increased when the optic nerve is involved, which can subsequently involve the subarachnoid space and spinal cord [12]. Bone metastases are the second most common site for metastases, with tubular bones being more commonly involved. Previous literature has reported rare cases of metastases to the mandible and contralateral orbit, with limited cases of soft tissue spread and parotid and submandibular gland involvement. Local spread can occur through direct contiguity with adjacent tissues and lymph nodes, and distant metastases are associated with vascular and lymphatic channels. Adjacent tissue invasion can also spread to the bone and the nasopharyngeal region via the sinus [13].

LCH is a rare disorder that primarily affects children and presents with one or more bone or skin abnormalities. Approximately 80% of patients experience bone lesions, while 40% have skin lesions [14], [15]. LCH can also involve soft tissues and the central nervous system, leading to mortality rates of 10–20% [16]. High-risk cases include those involving the liver, spleen, and bone marrow. The extent of LCH determines its classification as single-system or multi-system, with favorable outcomes generally observed in elderly patients and those with single-system LCH. Poor outcomes tend to occur in younger patients and those with multi-system LCH, lung involvement, or any other organ dysfunction [17]. Systemic chemotherapy has been found to be effective in pediatric patients with multi-system LCH in multiple randomized clinical trials [18].

When a metastatic disease is suspected, a comprehensive systemic workup should be performed, including brain and orbital MRI, lumbar puncture, CT abdomen, bone scan, and bone marrow biopsy with complete blood laboratory workup. MRI has a high sensitivity and specificity for diagnosing skull-based lesions and meningeal spread. The most definitive treatment for retinoblastoma is the removal of the eye before metastases occur. The extraocular disease is treated with chemotherapy and radiotherapy. Early diagnosis and treatment of retinoblastoma have a good prognosis, while patients with disseminated disease have a poor prognosis. Recent literature suggests that high-dose chemotherapy with autologous stem cell transplant can improve survival in these patients. Advanced cases may require radiotherapy and chemotherapy [19].

## Conclusions

This case report provides valuable insights into the management of a patient with both retinoblastoma and LCH. Our findings suggest that BRAF gene inhibitors may be effective in slowing down the progression of BRAF-positive LCH, and that standard drugs can also demonstrate significant efficacy when administered according to a well-planned treatment regimen. We recommend that children with skull swelling, positive family history, or unexplained visual loss undergo prompt ophthalmology review, followed by clinical follow-up. This case highlights the importance of a multidisciplinary approach to the management of complex diseases and underscores the need for further research to optimize treatment strategies for patients with multiple coexisting conditions.

## Notes

### Patient consent

Written and signed consent for the publication of this case report and accompanying data has been obtained from the guardian of the patient.

### Ethics statement

The manuscript complies with the ethical recommendations of the Declaration of Helsinki of the World Medical Association (WMA). The National Institute of Child Health’s radiology head of the department gave ethical approval for the publication of figures and the case.

### Author contributions

M.H, F.S, P.K.G contributed to the conception and design of the manuscript. M.H, P.K.G supervised the project. M.H, M.K.K, P.K.G provided the materials and contributed to data collection and processing. F.S, M.K.K, S.T contributed to interpretation and analysis of the project. F.S, M.K.K, S.T contributed to the literature review and writing of the manuscript, respectively. M.H, F.S, M.K.K, S.T, P.K.G. critically revised the manuscript. 

### Competing interests

The authors declare that they have no competing interests.
